# Comparison of Three Commonly Used Genetic Markers for Detection of *Leishmania Major*: An Experimental Study

**DOI:** 10.4314/ejhs.v31i4.6

**Published:** 2021-07

**Authors:** Hamed Behniafar, Vahideh Moin Vaziri, Seyyed Javad Seyyed Tabaei, Niloofar Taghipour

**Affiliations:** 1 Department of Medical Parasitology, Sarab Faculty of Medical Sciences, Sarab, Iran; 2 Department of Parasitology and Mycology, School of Medicine, Shahid Beheshti University of Medical Sciences, Tehran, Iran; 3 Department of Tissue engineering and Applied Cell Sciences, School of Advanced Technologies in Medicine, Shahid Beheshti University of Medical Sciences, Tehran, Iran

**Keywords:** Leishmania major, Molecular detection, kDNA, Cyt b, ITS1

## Abstract

**Background:**

Leishmaniasis is a vector-borne disease caused by an intracellular protozoan parasite called Leishmania spp. Different species produce different clinical outcomes; the majority of cases are cutaneous forms. Leishmania major is one of the main causative agents of cutaneous leishmaniasis (CL). Various methods are being using to diagnose CL, including microscopic examination, culture, and molecular detection of the parasite genome.

**Method:**

In the current study, we tried to compare three common molecular markers, including Kinetoplast DNA (kDNA), Cytochrome b (Cyt b), and Internal transcribed space 1 (ITS1), for the detection of Leishmania major. After cultivation of standard strain of L. major MHOM/IR/75/ER in RPMI 1640, certain number of promastigotes was subjected to DNA extraction and different PCR reactions.

**Results:**

The lowest number of the parasite (5 promastigotes) can be detected by kDNA-PCR, followed by Cyt b-PCR (10 promastigotes), and ITS1-PCR (50 promastigotes).

**Conclusion:**

In conclusion, kDNA-PCR was the most sensitive marker and may provide more reliable data in the initial screening, especially in false-negative results provided by parasitological methods due to the low number of parasites.

## Introduction

Leishmaniasis, as vector-borne disease, has been reported in 98 countries, including much of the Middle East countries (1). The causative agent of the disease is the *Leishmania* parasite, which has about thirty species (2,3). *Leishmania* parasites are dimorphic and found in amastigote form in the macrophage of the mammalian host and as flagellated promastigotes in the midgut of phlebotominae sand flies (4). The disease manifests three distinct clinical forms: cutaneous (usually self-limiting with a disfiguring scar), mucocutaneous and visceral, which could be fatal if left untreated (2,5,6). Early and accurate diagnosis and treatment will minimize the scar and the fatality rate of the disease. According to the World Health Organization, about 1 million cases of CL have been reported in the last five years (7). Diagnosis of cutaneous leishmaniasis based on the clinical signs is very complicated, but microscopic examination, serological tests and different molecular methods targeting the parasite genome have been used globally (3,8,9). The microscopical diagnosis was done by the observation of the amastigote forms in the demonstration of lesion scarification (8). This method has nearly 100% sensitivity, but it is dependent on the expertise of the microscopist and degree of parasitism (10).

Moreover, the parasite culture is not easy, failed frequently, and most of the time is not available in the laboratory in endemic areas (11). Although molecular methods have shortcomings such as high cost and limited application for field research, they are able to solve some of these mentioned problems (10, 12). Indeed, molecular methods are rapid, accurate, and minimally invasive diagnostic tests used vastly to detect *Leishmania* spp, by targeting different genes like kDNA, Cyt b, and ITS1 (9, 13–17). These three genes were selected because of their availability and widespread use in molecular studies.

When molecular methods were used for *Leishmania* detection, one of the most challenging issues is how a researcher can be sure that the negative results are trustworthy. This is not because of the less amount of DNA, lesser than the detection limit of the primers. This issue is especially important when a researcher works on the biological samples, which naturally consist of a low number of parasites like blood samples for VL diagnosis or in the gut of phlebotominae sandflies. For this purpose, we designed an experimental study to find a rough estimation of the minimum detectable amount of *Leishmania major* genome by commonly used primers for amplification of kDNA, Cyt b, and ITS1. In this study, we aimed to find the most sensitive genetic marker for the diagnosis of CL using conventional PCR.

## Material and Methods

**Preparation of promastigotes and DNA extraction**: It should be mentioned that this study was conducted at Shahid Beheshti University of Medical Sciences, Tehran, Iran, and from June to September 2019. The standard strain of *L. major* MHOM/IR/75/ER was obtained from the Department of Medical Parasitology, School of Public Health, Tehran University of Medical Sciences, Iran. Promastigotes were cultivated in RPMI 1640 (Gibco, Germany) medium with 20% FBS (Gibco) at 23 ºC. A certain number of promastigotes (500, 100, 50, 10, 5, and 1) was subjected to DNA extraction. The Neubauer chamber was used to count the parasite number. Isolation of one promastigote is very difficult, so we counted 10 promastigotes and diluted it 10 times. DNA was extracted from specified numbers of promastigotes using the DNG-plus extraction kit (SinaClon, Iran) following the manufacturer specification (1, 6, 18).

**PCR amplification**: Three mentioned fragments were amplified using following primers: 1) kDNA nested-PCR: CSB2XF (external-forward: CGAGTAGCAGAAACTCCCGTTCA), CSB1XR (external-reverse: ATTTTTCGCGATTTTCGCAGAACG), 13Z (internal-forward: ACTGGGGGTTGGTGTAAAATAG), LiR (internal reverse: TCGCAGAACGCCCCT) (16, 19); 2) ITS1-PCR: LITSR (forward): CTGGATCATTTTCCGATG, L5.8S (reverse): TGATACCACTTATCGCACTT (20–22); 3) Cyt b PCR: LCBF1 (forward: GTGTAGGTTTTAGTTTAGG), LCBR2 (reverse: CTACAATAAACAAATCATAATATACAAT T) (23, 24). The details of the PCR conditions have been shown in the Table 1. To evaluate the minimum detectable amount of DNA, several PCR reactions were carried out on different amount of DNA template yielded from different numbers of the parasite.

**Table 1:** PCR conditions and expected DNA fragment sizes for the amplification of kDNA, ITS1 and Cyt b in the current study

Type of PCR	PCR condition	Expected band (bp)
**kDNA-Nested-** **PCR**	Initial denaturation at 94 °C (5 min) **30 cycles**: [94 °C (60 s), 55 °C (60 s), 72 °C (60 s)] Final extension of 72 °C (5 min)	560
**ITS1-PCR**	Initial denaturation at 95 °C (5 min) **30 cycles**: [94 °C (30 s), 47 °C (30 s), 72 °C (60 s)] Final extension:72 °C (7 min)	360
**Cyt b PCR**	Initial denaturation at 94 °C (5 min) **39 cycles**: [94 °C (30 s), 50 °C (40 s), 72 °C (60 s)] Final extension of 72 °C (5 min)	866

Subsequently, 3 µl of each PCR product was electrophoresed at 55 V for 55 min in 1.5 % agarose gel and stained using ethidium bromide (EB). After that, PCR products were visualized and photographed using a GEL Imaging System (Bio-Rad Laboratories, CA, USA).

## Results

**Figure 1:**
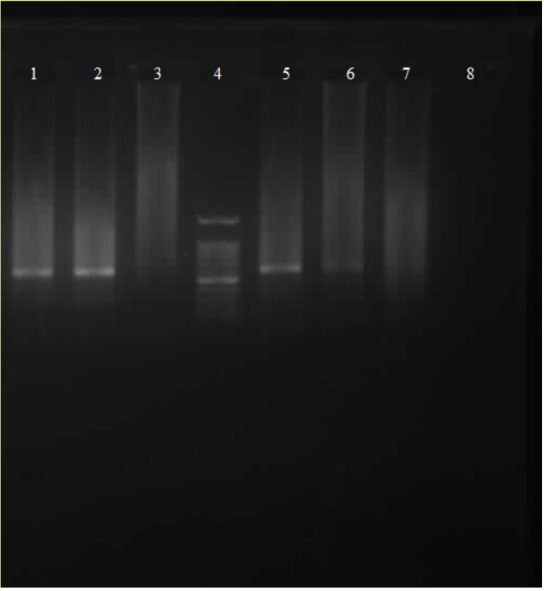
Agarose gel images of amplified Leishmania major kDNA gene fragments (about 560 bp) by using different amount of DNA templates. Lanes 1 and 2: PCR product of extracted DNA from 100 and 50 promastigotes, Lane 3: negative control (using both of external and internal primers), Lane 4: DNA ladder (100 bp, SinaClon, Iran), Lane 5, 6, 7: PCR product of extracted DNA from 10, 5 and 1 promastigotes, Lane 8: negative control (using internal primers).

**Figure 2:**
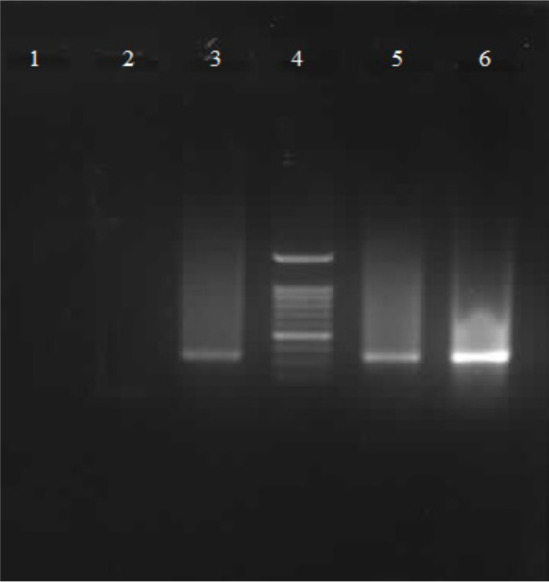
Agarose gel images of amplified Leishmania major ITS-1 gene fragments (about 360 bp) by using different amount of DNA templates. Lane 1: negative control, Lanes 2 and 3: PCR products of extracted DNA from 10 and 50 promastigotes, Lane 4: DNA ladder (100 bp, SinaClon, Iran), Lane 5 and 6: PCR product of extracted DNA from 100 and 500 promastigotes.

**Figure 3:**
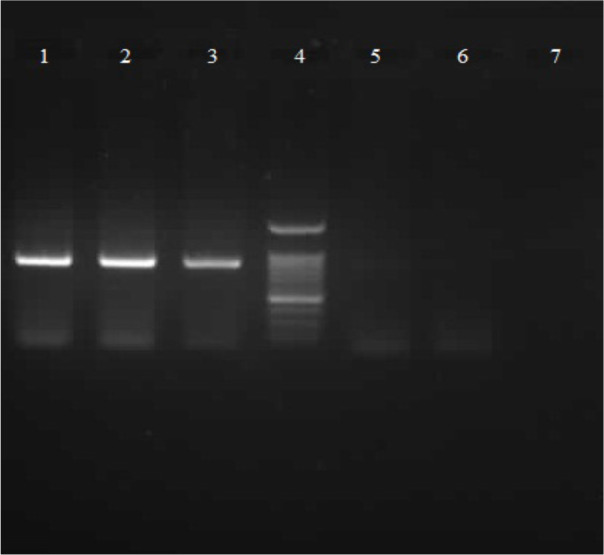
Agarose gel images of amplified Leishmania major Cyt b gene fragments (about 866 bp) by using different amount of DNA templates. Lanes 1 to 3: PCR product of extracted DNA from 500, 100 and 50 promastigotes, Lane 4: DNA ladder (100 bp, SinaClon, Iran), Lanes 5 and 6: PCR product of extracted DNA from 10, 5 promastigotes, and Lane 7: negative control.

PCR products were successfully amplified for three sets of primers. Negative and positive controls always monitored the reactions. In the current assay, kDNA-PCR, Cyt b-PCR, and ITS1-PCR could amplify *Leishmania* DNA samples, which were extracted from 5, 10, and 50 promastigotes, respectively (Figure 1 to 3). KDNA-PCR was the most sensitive diagnostic marker among those three and can detect 5 *Leishmania* promastigotes.

## Discussion

Human infection by *Leishmania* parasites can cause leishmaniasis, a complex of diseases which presents in many areas of tropical and subtropical countries. As different species of *Leishmania* are pathogenic to humans, the disease is polymorphic, and different clinical and epidemiological characteristics have been observed (25). Early and prompt diagnosis could enhance the chance of a better treatment (26). Recently, the most common methods for *Leishmania* detection in the biological samples are DNA-Based. These methods are also quite useful for species identification, which is also essential for better treatment.

However, a question raised in the mind is how a researcher can make sure that negative results in molecular assays could be trustable and not be due to the low amount of DNA, lesser than the minimum detectable limit of the PCR reactions? That is why we experimentally compare the minimum detection limit of the three main genetic markers commonly used in the laboratory to detect leishmaniasis.

Kinetoplast is a unique DNA in the mitochondrion of the *Leishmania* parasite, consisting of two parts, maxi-circle (present in a number of 30–50 copies/parasite, with 20–40 kb in length) and mini-circle kDNA (present in a number of 10,000–20,000 copies/parasite with 1 kb in length) (15). Based on the obtained results, kDNA-Nested-PCR was the most sensitive marker, as it can amplify DNA extracted just from 5 promastigotes. Having high copy numbers in parasite cells makes credit for that gene in *Leishmania* detection, most especially in the biological samples that contain a low amount of DNA (27,28). Moreover, kDNA can be used in discriminating *Leishmania* species, based on the size of the PCR products (16,28). On the other hand, the heterogenic nature of mini-circle networks has hampered the use of this genomic region for genetic variation (28), so in these case, this marker is not a right candidate.

Cyt b-PCR can amplify the DNA sample, which was extracted from 10 promastigotes, which gain second place in the tournament. Cytochrome *b* is the central redox catalytic subunit of quinol, which belongs to the mitochondrial genome. It encodes an enzyme involved in the mitochondrial respiratory chain by playing a role in the electron transport process (23).

The third place for amplification of *Leishmania* DNA is achieved by ITS1-PCR. Internal transcribed spacer I is a non-coding region of SSU-rRNA, which separates coding regions and placed between genes 18S and 5.8S. There are about 20–40 copies of SSU rRNA gene per cell in *Leishmania* species (29).

In conclusion, unlike the lower sensitivity of the last two methods compared to kDNA, they are better candidates to study the genetic variations(23, 29–31). Thus, kDNA-PCR may provide more reliable data in the initial screening, especially in false-negative results of *Leishmania* detection by the parasitological method. The limitation of this study was the impossibility of using clinical samples. It is not possible to accurately count the number of parasites in clinical specimens; just a qualitative guess is possible.
